# Basolateral Invasion and Trafficking of *Campylobacter jejuni* in Polarized Epithelial Cells

**DOI:** 10.1371/journal.pone.0054759

**Published:** 2013-01-28

**Authors:** Lieneke I. Bouwman, Paula Niewold, Jos P. M. van Putten

**Affiliations:** Department of Infectious Diseases and Immunology, Utrecht University, Utrecht, The Netherlands; University of Helsinki, Finland

## Abstract

*Campylobacter jejuni* is a major cause of bacterial diarrheal disease. Most enteropathogenic bacteria including *C. jejuni* can invade cultured eukaryotic cells via an actin- and/or microtubule-dependent and an energy-consuming uptake process. Recently, we identified a novel highly efficient *C. jejuni* invasion pathway that involves bacterial migration into the subcellular space of non-polarized epithelial cells (termed subvasion) followed by invasion from the cell basis. Here we report cellular requirements of this entry mechanism and the subsequent intracellular trafficking route of *C. jejuni* in polarized islands of Caco-2 intestinal epithelial cells. Advanced microscopy on infected cells revealed that *C. jejuni* invades the polarized intestinal cells via the subcellular invasion pathway. Remarkably, invasion was not blocked by the inhibitors of microtubule dynamics colchicine or paclitaxel, and was even enhanced after disruption of host cell actin filaments by cytochalasin D. Invasion also continued after dinitrophenol-induced cellular depletion of ATP, whereas this compound effectively inhibited the uptake of invasive *Escherichia coli*. Confocal microscopy demonstrated that intracellular *C. jejuni* resided in membrane-bound CD63-positive cellular compartments for up to 24 h. Establishment of a novel luciferase reporter-based bacterial viability assay, developed to overcome the limitations of the classical bacterial recovery assay, demonstrated that a subset of *C. jejuni* survived intracellularly for up to 48 h. Taken together, our results indicate that *C. jejuni* is able to actively invade polarized intestinal epithelial cells via a novel actin- and microtubule-independent mechanism and remains metabolically active in the intracellular niche for up to 48 hours.

## Introduction

*Campylobacter* is the most common cause of bacterial diarrheal disease worldwide [1]. It is estimated that each year up to 1% of the western population is infected with *Campylobacter* [2]. *Campylobacter jejuni* (*C. jejuni*) is the most prominent cause of human infections. Major infection sources are contaminated chicken and surface water. *C. jejuni* displays commensal behavior in chicken. The molecular basis of the difference in pathogenicity of *C. jejuni* in human and chicken still remains to be resolved. In the human intestine, *C. jejuni* penetrates the mucus and colonizes the intestinal crypts in a very efficient manner [3]. The crypts seem to be an optimal growth environment for *C. jejuni* [4]. Several studies suggest that after colonization, *C. jejuni* can cross the mucosal barrier and invade intestinal cells [5]–[8]. The exact mechanism(s) of invasion and the intracellular processing of the bacteria are not well understood.

Experimental studies using cell culture models indicate that *C. jejuni* can enter cells via different routes. Both actin-dependent and microtubule-dependent uptake into eukaryotic cells have been reported [7]–[11]. The uptake process may require cellular factors such as caveolin-1 and the small Rho GTPases Rac1 and Cdc42, but not dynamin [12]–[14]. The reports of different uptake requirements suggest that *C. jejuni* has evolved multiple mechanisms to gain access to eukaryotic cells, albeit with variably efficiency [8], [15]. One of the most effective invasion pathways resulting in nearly 100% of bacterial uptake at low inocula, involves the subvasion entry pathway. This mechanism involves migration of *C. jejuni* underneath cultured cells, followed by bacterial invasion from the basal cell side instead of the apical side [17]. The sequence of events that drive this uptake process remains to be resolved.

Once inside the eukaryotic cells, *C. jejuni* is generally assumed to reside within a membrane-bound compartment. Both localization in endolysosomal compartments as well as in so-called *Campylobacter* containing vacuoles (CCV) have been reported [14]. CCV are supposed to be a special compartment specifically induced by *C. jejuni*, reminiscent of *Salmonella* that creates its own vacuole *Salmonella* containing vacuole SCV (for review: see [16]). Whether *C. jejuni* survives inside epithelial cells is still under investigation [14], [17]. Intracellular survival may vary dependent on the nature of the *C. jejuni* containing compartment. Furthermore, the procedure to recover the intracellular *C. jejuni* may influence bacterial survival assay results [14], [17], [18].

The present study was designed to determine the unknown molecular events that are at the basis of the recently discovered *C. jejuni* subvasion entry route and to determine the trafficking and survival of *C. jejuni* after use of this infection pathway. Experiments were performed using polarized Caco-2 intestinal epithelial cells as a model system. A novel luciferase reporter system was developed to determine intracellular survival without the need of the debated bacterial recovery procedure. Our results indicate that the *C. jejuni* subvasion entry mechanism is driven by a novel actin- and microtubule-independent process that results in high numbers of intracellular membrane-bound bacteria of which a subset survives for up to 48 hours.

## Results

### Basalateral C. jejuni invasion of polarized epithelial cells

**Figure 1:**
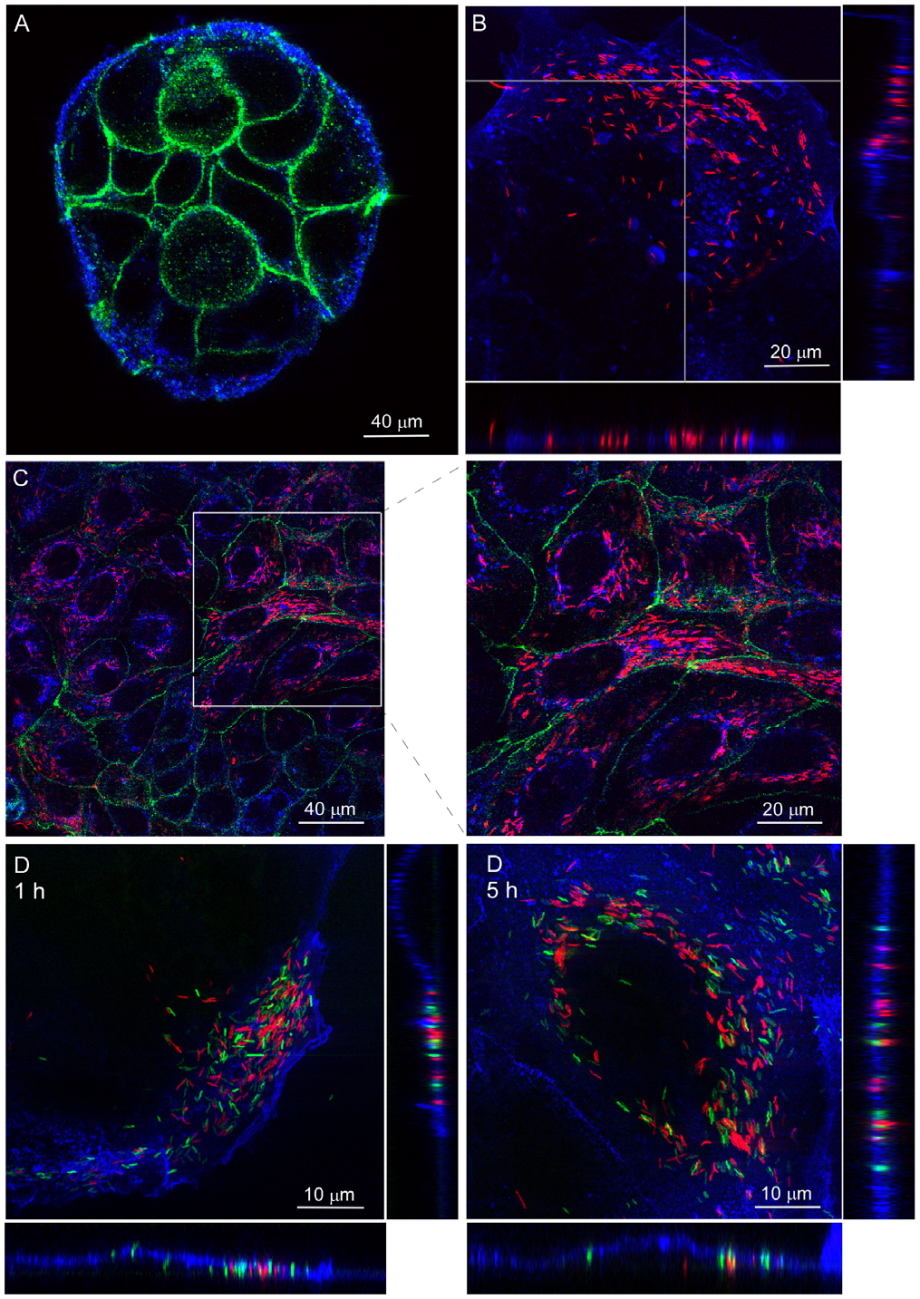
*C. jejuni* invades polarized Caco-2 islands via subvasion with high efficiency. Confocal laser microscopy on non-infected and *C. jejuni*-infected islands of polarized Caco-2 cells. (A) Uninfected island of Caco-2 cells stained with the membrane marker WGA-Alexa fluor633 (Blue) and an anti-occludin antibody (Green) showing the presence of tight junctions. (B) Caco-2 cells (Blue) at 1 h of infection in DMEM showing *C. jejuni* strain 108p4 (Red) mostly located at the basal side of cells near the edge of the island of polarized cells. (C) Caco-2 cells (Blue) at 5 h of infection in DMEM demonstrating intracellular *C. jejuni* strain 108p4 (Red) at the center of the island of cells with tight junctions (Green). (D). Polarized Caco-2 cells (Blue) infected (1 h and 5 h) with a mixture of *C. jejuni* strains 108p4 (Red) and 81–176 (Green) showing invasion of Caco-2 cells by both strains. Transversal optical sections of the cells are depicted at the bottom of each panel to show the location of the bacteria relative to the cell basis.

*C. jejuni* poorly enters intact monolayers of polarized epithelial cells but invades non-polarized epithelial cells from the basolateral cell surface [19]–[22]. To investigate the ability of *C. jejuni* to invade polarized epithelial cells from the basal cell side, we performed infection assays with cultured islands of polarized Caco-2 intestinal cells. This approach was meant to enable the bacteria to access the subcellular compartment from the edges of the island and from there to migrate underneath the cells to invade polarized cells without chemical disruption of tight junctions. The presence of tight junctions in the cultured islands of Caco-2 cells was confirmed by confocal microscopy on occludin-stained cell cultures (Figure 1A) and considered as indicator of cell polarization.

Microscopic examination on islands of polarized Caco-2 cells infected with mCherry-fluorescent *C. jejuni* strain 108 for 1 h showed *C. jejuni* mainly located underneath cells at the edges of the islands (Figure 1B). Confocal microscopy at 5 h of infection revealed the presence of large numbers of intracellular *C. jejuni* not only in cells at the edge of the islands but also in the center (Figure 1C). These observations reflect the recently discovered migration of *C. jejuni* underneath non-polarized cells (subvasion) which is followed by bacterial invasion from the basal cell side [19]. Similar subvasion-dependent invasion of polarized Caco-2 islands was observed for the GFP-fluorescent 81–176 (Figure 1D). Control experiments with intact monolayers of Caco-2 cells rather than islands of cells yielded no invasive *C. jejuni* consistent with previous results that showed minimal bacterial penetration via the apical cell surface [19]. Together, these results suggest that *C. jejuni* efficiently invades an intact layer of polarized epithelial cells from the basal cell side once an access point is available.

### Actin and microtubule-independent C. jejuni invasion of polarized Caco-2 cells

**Figure 2:**
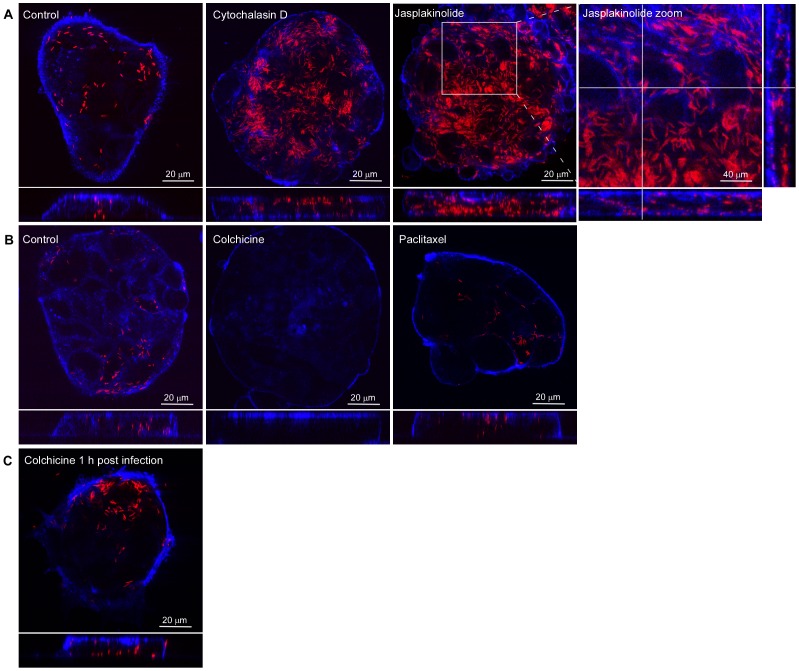
Microtubule and actin cytoskeleton-independent *C. jejuni* invasion of Caco-2 cells. Islands of polarized Caco-2 cells were infected (2 h) with *C. jejuni* strain 108p4 (Red) in the absence of presence of the indicated actin cytoskeleton or microtubules disrupting or stabilizing drugs. Cells were fixed and stained with WGA-alexa fluor633 (Blue). Infected cells were visualized with confocal microscopy. The following drugs were used: (A) cytochalasin D (3 µM) and jasplakinolide (1 µM) added at 1 h prior to infection; (B) colchicine (10 µM) or paclitaxel (1 µM) added at 1 h prior to infection; (C) Colchicine (10 µM) added at 1 h after start of the infection and cells fixed at 2 h post infection. As control, cells were pre-treated with an equivalent amount of solvent DMSO (Final concentration 0.2%).

As a first step towards understanding the mechanism(s) driving the basal invasion of the polarized cells, the islands of cells were incubated with the actin filament disrupting or stabilizing agents cytochalasin D and jasplakinolide, or the microtubule-filament disrupting or stabilizing drugs colchicine and paclitaxel. The compounds were added to the epithelial cells at 1 h prior to inoculation of *C. jejuni* strain 108. Disruption of the actin cytoskeleton dynamics using cytochalasin D (3 µM) or jasplakinolide (1 µM) enhanced rather than blocked *C. jejuni* invasion, as evident from the number of intracellular bacteria observed in the confocal microscope (Figure 2A). Similarly, fixation of the microtubules with paclitaxel (1 µM) did not inhibit *C. jejuni* invasion (Figure 2B). Disruption of the microtubules with colchicine (10 µM) severely reduced the number of intracellular bacteria (Figure 2B), but also the number of subcellular *C. jejuni*. In an attempt to distinguish the effect(s) of colchicine on the subvasion and subsequent invasion process, the islands of polarized epithelial cells were infected for 1 h with *C. jejuni* 108 to allow bacterial subvasion to occur, prior to the addition of colchicine. This procedure did not block bacterial invasion (Figure 2C), suggesting a role of microtubules in allowing subcellular migration rather than bacterial invasion.

**Figure 3:**
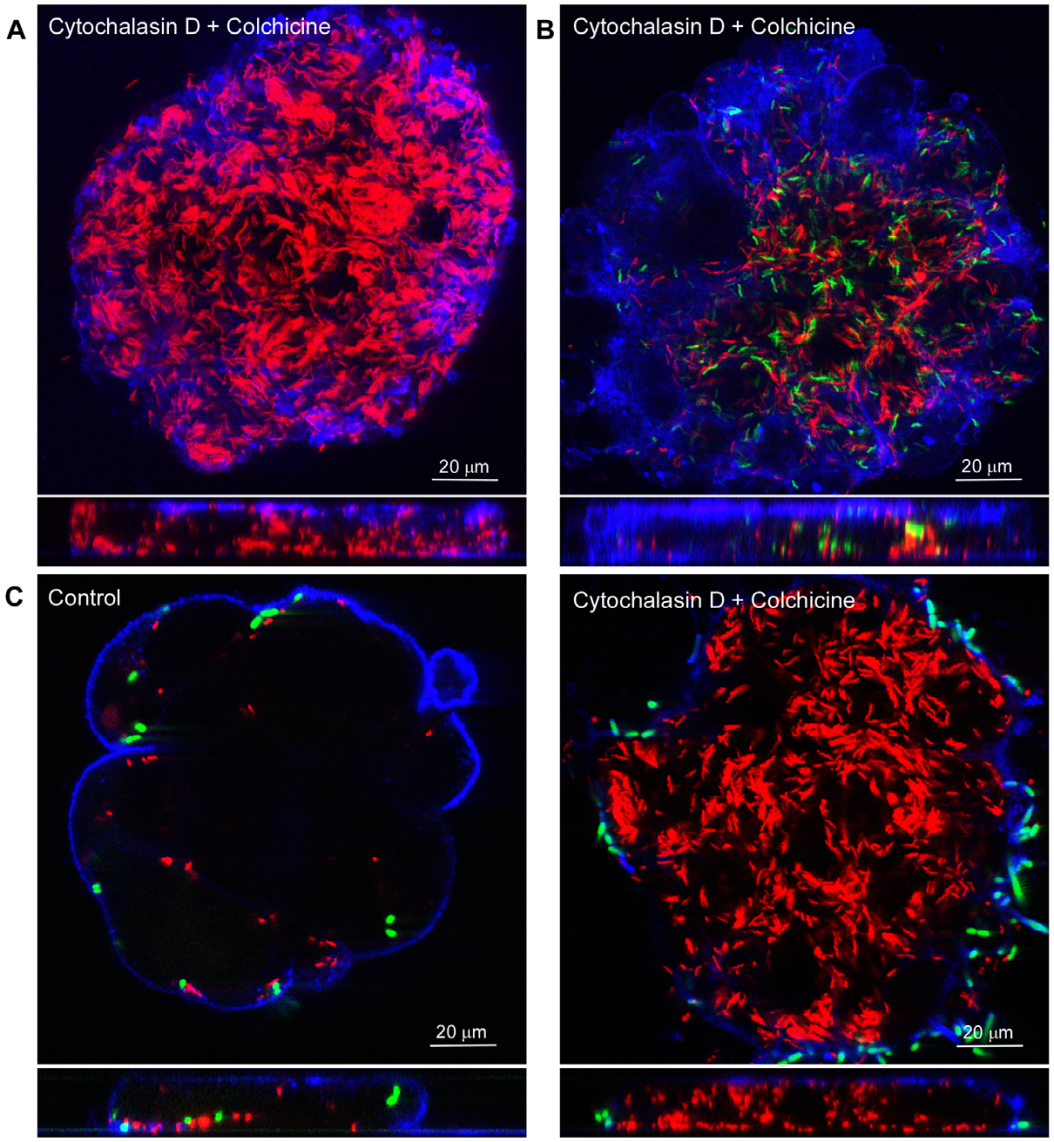
Enhanced invasion of *C. jejuni* strains 108 and 81–176 after disruption of the host cell cytoskeleton. Islands of polarized Caco-2 cells were pre-treated (1 h) with cytochalasin D (3 µM) and colchicine (10 µM) and infected (2 h) with *C. jejuni* and/or *E.coli^inv^* (Green). Cells were fixed and stained with WGA-alexa fluor633 (Blue). Infected cells were visualized with confocal microscopy. Polarized cells were infected with: (A) *C. jejuni* strain 108p4 (B) *C. jejuni* strain 108p4 (Red) and 81–176 (Green) in Hepes buffer. (C) *C. jejuni* strain 108p4 (Red) and *E. coli^inv^* (Green). As control, cells were pre-treated with an equivalent amount of solvent DMSO (Final concentration 0.2%). Note the strong invasion of *C. jejuni* and the inhibition of *E. coli^inv^* invasion in the presence of the added compounds.

To corroborate the actin and microtubule-independent invasion of *C. jejuni*, the polarized Caco-2 cells were treated with the combination of cytochalasin D (3 µM) and colchicine (10 µM) prior to infection with *C. jejuni* strain 108 or strain 81–176. This yielded large numbers of intracellular bacteria for both strains (Figure 3A and 3B). Control experiments in which Caco-2 polarized islands or semi-confluent int-407 (Figure S1) were co-infected with a mixture of *C. jejuni* 108 and invasive *E. coli* expressing the invasin of *Y. pseudotuberculosis (E. coli^inv^*), demonstrated the expected invasion of *C. jejuni* (Figure 3C), whereas the uptake of *E. coli^inv^* was effectively blocked, in agreement with the well-documented actin-dependence of the *E. coli^inv^* uptake mechanism [23]. Overall, the results suggest that *C. jejuni* subvasion requires intact microtubules, but that efficient *C. jejuni* invasion into polarized epithelial cells can occur via an actin- and microtubule-independent mechanism.

### C. jejuni invasion in ATP-depleted epithelial cells

**Figure 4:**
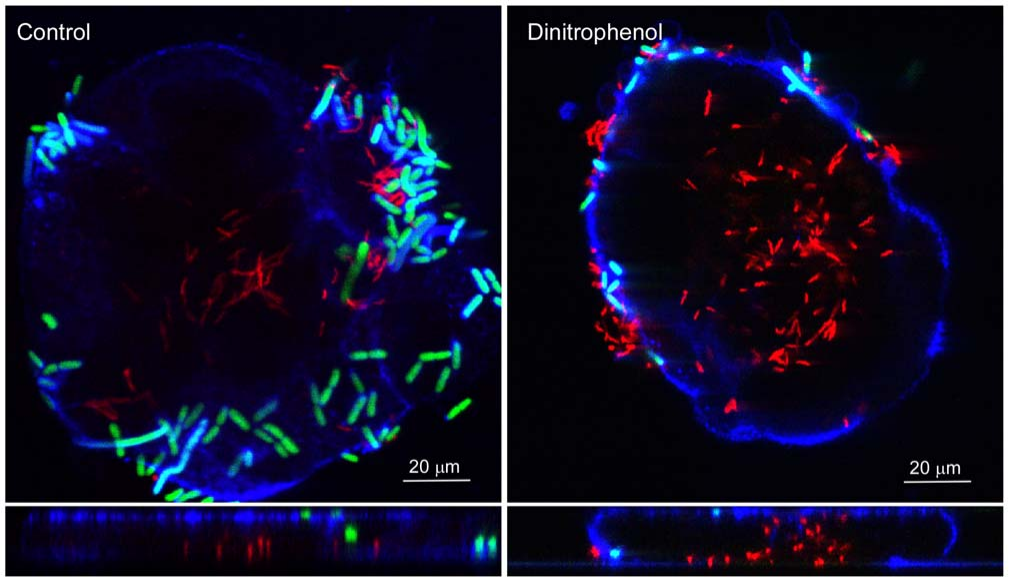
Effect of ATP depletion on *C. jejuni* and *E. coli^inv^* invasion. Islands of polarized Caco-2 cells were treated for 1 h with 3 mM of DNP and then infected with *C. jejuni* strain 108p4 (Red) and *E. coli^inv^* (Green) for 2 h after which the cells were stained with WGA-Alexa fluor633 (Blue), fixed, and visualized with confocal microscopy. As a control, islands were treated with an equivalent amount of solvent acetone (final concentration: 0.3%) and infected. Note that DNP inhibits the invasion of *E. coli^inv^* but not of *C. jejuni*.

Most enteropathogens trigger their own uptake into eukaryotic cells through activation of cellular endocytic processes that require energy-consuming rearrangement of the actin cytoskeleton and/or microtubule network [24], [25]. As *C. jejuni* apparently can invade eukaryotic cells via an actin- and microtubule-independent pathway, we determined the energy dependence of the *C. jejuni* invasion mechanism. Hereto infection experiments were performed in the presence of 3 mM of 2,3-dinitrophenol (DNP). DNP disrupts the production of mitochondrial ATP causing depletion of most of the cellular ATP. DNP treatment inhibits *Salmonella* and *Shigella* invasion by more than 95% [26]. Confocal microscopy on polarized Caco-2 cells infected (2 h) with a mixture of GFP-fluorescent *E. coli^inv^* and mCherry fluorescent *C. jejuni* strain 108 demonstrated the expected severe reduction of invasion of the DNP-treated cells for *E. coli^inv^* (Figure 4). However, *C. jejuni* still invaded the polarized cells in the presence of DNP. This result is consistent with the apparent absence of energy-consuming cytoskeletal changes during the *C. jejuni* entry process.

### C. jejuni traffics into an endolysosomal compartment

**Figure 5:**
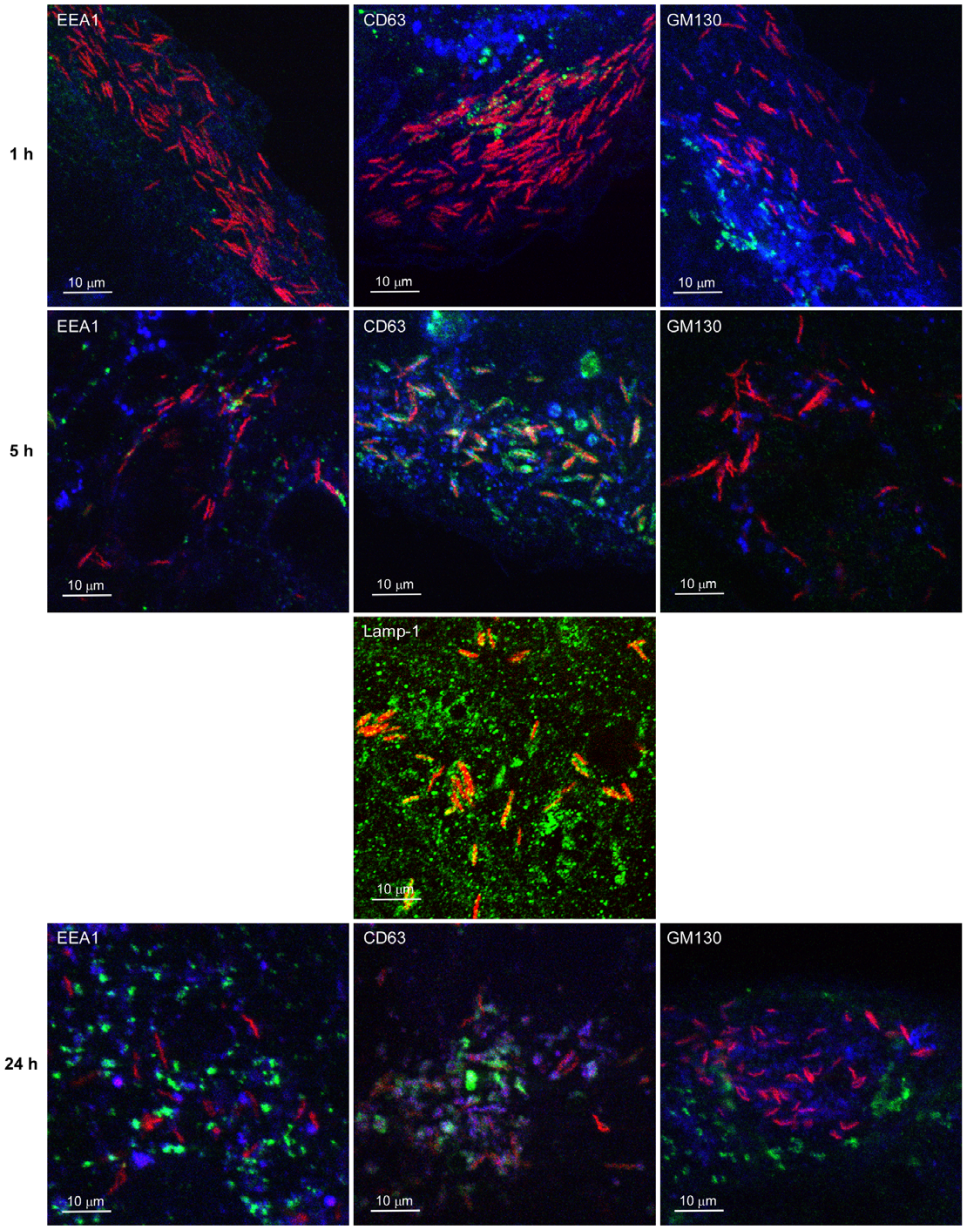
*C. jejuni* resides within CD63-positive cellular compartments. Islands of Caco-2 were infected with *C. jejuni* strain 108 (red) for 1 h, washed, further incubated for up to 24 h, and fixed. After fixation, the cell surface was stained with WGA-Alexa fluor633 (Blue) and, after permeabilization, cellular compartments (Green) were stained with the marker antibodies EEA1 (early endosome), CD63 and Lamp-1 (endolysosome), or GM130 (Golgi apparatus) in combination with goat anti-mouse-Alexa fluor488. Infected cells were visualized with confocal microscopy. Note the strong co-localization between invasive *C. jejuni* and the CD63-positive compartment at 5 h of infection.

To learn more about the trafficking of *C. jejuni* that entered the polarized epithelial cells via the basal invasion pathway, we followed the intracellular localization of the bacteria using confocal laser microscopy. To synchronize bacterial invasion into the polarized Caco-2 islands, the cells were infected with mCherry-fluorescent *C. jejuni* strain 108 for 1 h to allow subvasion to occur, after which the medium was replaced to remove all extracellular bacteria limiting further subvasion. At different duration of infection, the cells were fixed and immuno-stained using EEA1, CD63 and GM130 as markers of early endosomes, late endo(lyso)somes, and Golgi apparatus, respectively. Confocal microscopy showed that after 1 h of infection *C. jejuni* did not co-localize with any of the labeled cellular compartments and were mainly present at the basal cell surface (Figure 5 and Figure S2). After 5 h of incubation, the majority (∼95%) of the *C. jejuni* resided in CD63-positive membrane-bound vacuoles. *C. jejuni* remained in these compartments for the duration of the infection (24 h). Similar co-localization was observed with the late endosomal marker Lamp-1 (Figure 5) consistent with earlier studies [14], [27]. At the times of infection (1h, 5h, 24 h) investigated *C. jejuni* only rarely co-localized with EEA1-positive early endosomal compartments and did not seem to be specifically localized in close vicinity of the Golgi apparatus. After 48 h of infection, mCherry-positive bacteria did appear absent from the cells. However, staining with anti-*C. jejuni* antibody targeting the outer membrane still revealed the presence of intracellular *C. jejuni*, although these bacteria had lost their characteristic spiral shape (Figure S3).

### Intracellular survival of a subset of C. jejuni

**Figure 6:**
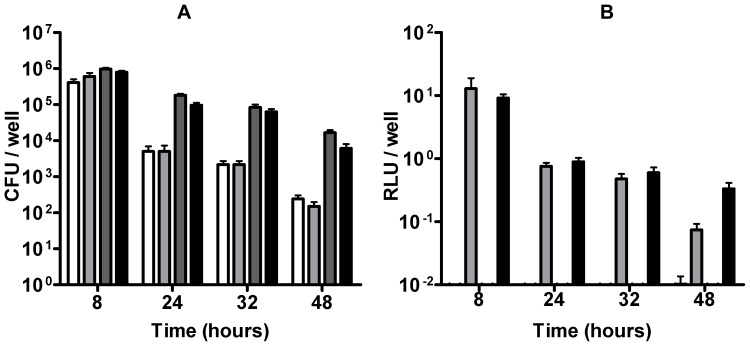
Bacterial viability of intracellular *C. jejuni*. Islands of polarized Caco-2 cells were infected with *C. jejuni* for 5 h in Hepes buffer, washed, incubated (3 h) with gentamicin (250 ìg/ml) in DMEM, washed again, and incubated for an additional 42 h in DMEM plus 10% FCS with a low dose of gentamicin (50 ìg/ml). At the indicated times, samples were prepared for bacterial viability assay. (A) Gentamicin killing assay showing the bacterial recovery of intracellular *C. jejuni* strains 108 containing pMA5-metK-luc (white and light grey bars) and 81–176 containing pMA5-metK-luc (dark grey and black bars) from Caco-2 cells at the indicated duration of infection. CFU were enumerated after 48 h of recovery on agar plates in a 0.2% oxygen (white and dark grey bars) and 5% oxygen (light grey and black bars) environment and indicated as CFU per well. (B) Bacterial viability as measured by bacterial luciferase reporter assay at the indicated time points. Values for results presented in (A) and (B) are the mean ± SEM of at 3 independent experiments in performed in duplicate.

The intracellular fate of the large numbers of *C. jejuni* that invaded via the basal invasion pathway was investigated first using the gentamicin recovery assay. Polarized Caco-2 islands were infected with *C. jejuni* strains 108 or 81–176 for 5 h, treated with gentamicin (250 µg/ml) for 3 h, and then lysed directly or at various time points after further incubation of the cells in the presence of a low dose (50 µg/ml) of gentamicin. Intracellular bacteria were recovered on saponin agar plates at 5% O_2_ or 0.2% O_2_, at different time points during infection (Figure 6A). After 8 h of infection about 10^6^ intracellular bacteria per well were recovered for both strains. At prolonged infection (24–48 h), bacterial numbers gradually declined. For this period, *C. jejuni* strain 81–176 showed higher recovery rates than strain 108. After 48 hours, a small subpopulation of *C. jejuni* was still successfully recovered from the cells. Recovery at different oxygen levels which may facilitate the bacterial transition to the extracellular environment [14], had no significant effect on the results, although slightly more bacteria were recovered in a low oxygen environment at the later time points.

As the gentamicin assay results are the end product of bacterial invasion, intracellular survival and the bacterial adaptive abilities during recovery onto agar plates, we designed a novel luciferase reporter assay that allows direct assessment of intracellular bacterial viability. To establish this assay, the firefly luciferase reporter gene *of Photinus pyralis* was placed behind the *C. jejuni metK* promoter and cloned into the conjugative plasmid pMA5. The plasmid was then introduced into *C. jejuni* strains 108 and 81–176. The used *Photinus pyralis* gene encodes a luciferase with short half-life and high sensitivity [28], enabling the detection of approximately 10^5^ bacteria. The half-life of the luciferase in *C. jejuni* was determined to be about 30 min (Figure S4). Microscopy on infected cells using the recombinant *C. jejuni* revealed similar bacterial invasion levels as the parent strains at 3 h of infection. Luciferase assays on infected cells showed that luciferase values gradually dropped between 24 – 48 h of infection. After 48 h luciferase values were still detected. This time no difference was noted between the two strains, as comparable numbers of bacteria for both strains were measured at 8–32 h of infection, in contrast to the results of the gentamicin assay (Figure 6B). These results suggest that invasion of different *C. jejuni* strains via the subvasion entry pathways results in a subset of *C. jejuni* that remains metabolically active inside the eukaryotic cells for up to 48 h.

## Discussion

Bacterial pathogens are generally assumed to enter mucosal cells via microbe-directed uptake mechanisms that involve an energy-consuming rearrangement of the host cell cytoskeleton [29]-[32]. Here we report that different strains of *C. jejuni* efficiently invade polarized epithelial cells via an actin- and microtubule-independent mechanism, even in the presence of the ATP-depleting compound DNP. The highly efficient invasion occurred at the basal cell side of polarized epithelial cells and resulted in intracellular bacteria residing in CD63-positive cellular compartments. A novel luciferase reporter-based bacterial viability assay revealed survival of a subset of the intracellular *C. jejuni* for up to 48 hours. The unusual qualities of the described *C. jejuni* invasion mechanism underline the different nature of this pathogen compared to other enteropathogens.

*C. jejuni* invasion of eukaryotic cells is well established. Multiple uptake pathways have been proposed and frequently shown to depend on actin- and/or microtubule rearrangements in the host cell (for reviews, see [33]–[35]). Our results indicate that the highly efficient *C. jejuni* entry of the polarized cells involves a different mechanism that does not require gross rearrangement of the cytoskeleton. In fact, most efficient entry of *C. jejuni* was noted in the presence of the actin- and microtubule polymerization inhibitors. The increase in *C. jejuni* invasion in the presence of cytochalasin D may be explained by increased accessibility to the subcellular space due to retraction of cell protrusions. The actin cytoskeleton stabilizing compound jasplakinolide also increased invasion, probably due to inhibition of the turnover of actin filaments which may eventually also result in a loosening of cell attachment. At first glance, the microtubule inhibitor colchicine seemed to block invasion. However, when added together with cytochalasin D, or at one hour after inoculation, this effect was overcome suggesting that colchicine merely prevented subvasion rather than the bacterial entry into the cells. We are not aware of other enteropathogens capable of entering mucosal cells via a seemingly actin- and microtubule-independent pathway. One possible scenario is that *C. jejuni* actively penetrates the host cells driven by flagella motility perhaps to some extent reminiscent of actin motility-driven cell to cell movement of intracellular *Listeria monocytogenes* to neighboring cells [36]. This hypothesis would be consistent with the limited effect of ATP depletion of the host cells on the *C. jejuni* invasion compared to the strong inhibition of *E. coli^inv^* invasion (Figure 3) and *Salmonella* and *Shigella* invasion [26]. We consider a differential effect of DNP on *E. coli* and *C. jejuni* as a cause of the different invasion unlikely as invasin-mediated uptake does not require viable or motile bacteria; even invasin-coated beads are taken up by eukaryotic cells [37]. The observed negative effect of DNP on *C. jejuni* motility was overcome after the addition to the medium of alternative electron acceptors.

The efficient invasion of *C. jejuni* into polarized Caco-2 cells resembles the previously reported subcellular route of invasion of the pathogen into non-polarized cells [19]. For the entry of polarized epithelial cells it was essential to use islands of polarized epithelial cells rather than intact monolayers. *C. jejuni* has been reported to traverse intact monolayers [20], [38], [39]. In the current study, we never observed bacterial entry of intact monolayers from the basal cellular compartment. Apparently, a port of entry to gain access to the subcellular compartment, as was present at the edges of the polarized Caco-2 islands, is required to start the cellular infection. This is in line with previous work showing *C. jejuni* infection of polarized cells only after EDTA-induced disruption of the tight junctions which provide access to the subcellular space [19], [22]. This raises the question as to how *C. jejuni* may reach the subcellular space and invade the cells when the mucosal barrier is intact. Here basically three scenarios can be envisioned. One possibility is that *C. jejuni* transiently disrupts or translocates across tight junctions to pass the epithelial cell layer. This has been reported to occur *in vitro* for cells grown on a Transwell support [40]–[42]. Alternatively, the growth of *C. jejuni* in the intestinal crypts may ultimately disrupt the integrity of the epithelial cells, resulting in damage to the cell barrier and free access to the subcellular space. Finally, *C. jejuni* may target and passage through to mucosal M-cells, which are specialized epithelial cells equipped to sample the intestinal lumen and to deliver the content to underlying immune cells. M-cell mediated transcytosis to the subcellular compartment and subsequent entry into epithelial cells has been demonstrated to occur *in vivo* for enteropathogens like *Shigella* (for reviews: see [43], [44]). Interaction of *C. jejuni* with M-cells has been observed in the rabbit intestine model [45].

The *C. jejuni* invasion at the cell basis of polarized epithelial cells ultimately resulted in the presence of *C. jejuni* in Lamp-1 and CD63-positive membrane-bound compartments (Figure 4). This localization resembles the endolysosomal compartment that has been identified in several other studies as an intracellular niche for *C. jejuni*, despite that these bacteria may have been internalized via a different uptake system [14], [18]. The apparent absence of co-localization of *C. jejuni* with the early endosome marker EEA1 may indicate a rapid intracellular trafficking or perhaps even a bypassing of this route after uptake by this novel invasion pathway. At prolonged infection, a close association of *C. jejuni* with the Golgi apparatus has been reported [14]. Although the same strain was used in the present study, we were not able to confirm this finding, but we feel it too early to conclude that this variable result is caused by *C. jejuni* using different invasion pathways. An unexpected microscopic observation was the apparent absence of *C. jejuni* in the host cells after 48 h of infection. Control experiments using *C. jejuni*-specific antibodies revealed the presence of *C. jejuni* albeit with changed morphology. The loss of the bacterial fluorescent marker (mCherry protein) may indicate that *C. jejuni* underwent major metabolic changes at prolonged infection [46].

Classical gentamicin killing assays confirmed the presence of viable *C. jejuni* inside the polarized epithelial cells. The number of bacteria gradually declined with duration of the infection with a slight difference in recovery between the two tested *C. jejuni* strains (Figure 6B). At this point, it should be noted that the gentamicin killing assay reflects the ability of *C. jejuni* to invade cells and to adapt to the intracellular environment and, subsequently, to the extracellular environment during the recovery on agar plates. It has previously been demonstrated that efficacy of the *C. jejuni* recovery from the intracellular compartment varies with the growth conditions during recovery [14], [17]. To corroborate our findings we therefore developed a novel complementary bacterial viability assay based on the production of firefly luciferase with a short half-life. Introduction of a plasmid carrying the luciferase gene of *Photinus pyralis* in front of the metK promoter into *C. jejuni* enabled assessment of bacterial viability with a sensitivity of 10^5^ bacteria. Previous proteome analysis of intracellular *C. jejuni* has shown stable levels of the methionine adenosyltransferase transcribed by the *metK* gene [46]. The application of this novel method learned that the difference in bacterial 'survival' measured for the two strains in the gentamicin assay was, in fact, caused by a difference in the efficiency of *C. jejuni* recovery from the cells. As important, the combination of both methods demonstrated that a subset of the intracellular *C. jejuni* population was still surviving and metabolically active after 48 h of infection, suggesting that they succeeded in adopting an intracellular lifestyle.

In conclusion, our results demonstrate that *C. jejuni* has the ability to invade polarized epithelial cells via an actin- and microtubule independent process, even after depletion of host cell ATP and that a subset of intracellular bacteria can survive intracellularly for prolonged periods. Major future challenges are to demonstrate the activity of this novel infection route in the natural environment of the human host and to develop inhibitors of infection.

### Materials and Methods

#### Cell culture and reagents

Caco-2 cells (CRL 2102, ATCC) were routinely cultured in 25 cm^2^ flask in 6 ml of DMEM 10% FCS+non-essential amino acids (NAA) at 37°C and 5% CO_2_. The following reagents were used: cytochalasin D, colchicine, paclitaxel, 2,3-dinitrophenol (DNP), and NAA (Sigma); Jasplakinolide and fluorsave (Calbiochem); Mouse anti-EEA1 and mouse anti-GM130 (Becton Dickinson); Mouse anti-occludin, phalloidin-Alexa fluor633, WGA-Alexa fluor633, and the secondary antibodies goat anti-mouse-Alexa fluor488, goat anti-mouse-Alexa fluor568 and goat anti-rabbit-Alexa fluor568 (Invitrogen). Mouse anti-CD63 (Immunotech); Rabbit anti-Lamp-1 (ab24710) (Abcam); Reporter Lysis Buffer (RLB) and Luciferase Assay Agent (Promega); Saponin agar plates, Mueller Hinton plates, Heart Infusion (HI) plates, HI broth (Biotrading), and CCDA (SR0155) (Oxoid). The *C. jejuni*-reactive polyclonal antiserum 625 was made by Eurogentec via immunization of rabbits with the outer membrane fraction of *C. jejuni* strain 81116.

#### Bacterial culture

*C. jejuni* strain 108 (wild type and the hyperinvasive p4 variant) [17] and strain 81–176 [47] were routinely grown under microaerophilic conditions at 37°C on saponin agar plates containing 4% lysed horse blood or in 5 ml of HI broth (160 rpm). Kanamycin (50 µg/ml) was added to the media when appropriate. The GFP-fluorescent invasive *E. coli* strain *E. coli^inv^* [23] was grown on Luria-Bertani (LB) agar plates or in 5 ml of LB broth containing kanamycin (50 µg/ml) and chloramphenicol (40 µg/ml) at 37°C in air.

#### Infection assay

Caco-2 cells were grown on 12 mm circular glass slides for 48 h in DMEM+10% FCS+NAA at 37°C in a 10% CO_2_ atmosphere. Caco-2 cell islands were considered to consist of polarized cells when tight junctions were present as evident from occludin staining. All infection assays were performed as described here unless indicated otherwise. Cells were rinsed twice and incubated in 1 ml of pre-warmed Hepes buffer saline [48] containing 2 mM of phosphate until further use. In all infection assays, *C. jejuni* strains expressing the fluorescent markers mCherry or green fluorescent protein (GFP) were used, unless indicated otherwise. Bacteria were grown (16 h, 37^o^C) in HI broth, collected by centrifugation (10 min, 3,000 *x g*), resuspended in pre-warmed Hepes buffer, and added to the cells at a bacteria to host cell ratio of 200. After 1 h of incubation (37°C, 5% CO_2_), the extracellular bacteria were removed by rinsing the cells twice with 1 ml of Dulbecco's PBS (DPBS). The washed cells were further incubated in 1 ml of fresh DMEM without FCS for the duration of the assay. In co-infection experiments *E. coli^inv^* was added at a bacteria to host cell ratio of 20. The potential infection inhibitors cytochalasin D, colchicine, paclitaxel, jasplakinolide, 2,3-dinitrophenol (DNP) or a combination of these compounds were added at 1 h prior to the addition of the bacteria, unless indicated otherwise. When potential infection inhibitors were used, the infection assays were performed in DMEM. When DNP was used, 50 mM of fumaric acid and 50 mM of sodium nitrate were added to the medium (DMEM) as alternative electron acceptors in order to ensure bacterial motility was maintained. DNP (3 mM) reduced ATP levels in Caco-2 cells by >70% as measured by the ATP determination kit (Invitrogen). Infections were stopped by rinsing the cells with 1 ml of DPBS and then used in survival assays or prepared for microscopy.

#### Confocal microscopy

Cells were processed for confocal microscopy as previously described [49] with some minor modifications. Infected Caco-2 cells were rinsed once with DPBS and the cell membrane was stained (10 min, 37°C, 5% CO_2_) with WGA-Alexa fluor633 (1:500 dilution) in DPBS prior or post fixation. Samples were washed three times with DPBS, rinsed with 2% paraformaldehyde (PFA) in 100 mM of phosphate buffer (pH 7.4), fixed by incubating (1 h) with 4% PFA in 100 mM of phosphate buffer (pH 7.4), and when needed, permeabilized by incubating (30 min) with 1% Triton X−100+1% BSA in DPBS. Cell organelles and (when needed) intracellular *C. jejuni*, were stained by an incubation (1 h, 20°C) of the cells with the appropriate primary antibody in DPBS+2% BSA and, subsequently, with the appropriate secondary antibody (1:100) in DPBS+2% BSA. After three rinses with 1 ml of DPBS, the stained samples were mounted in fluorsave. Specimens were viewed in a Bio-Rad radiance 2100MP multiphoton confocal laser microscope and analyzed using ImageJ software. Islands of Caco-2 cells consisted of approximately 20 cells as can be deduced from Fig. 1. Per experiment the entire slide was examined and on average three representative islands of cells were imaged by making Z-stacks of the entire island. This did not allow a reliable microscopic quantification of the number of intracellular bacteria.

#### Construction of fluorescent C. jejuni and E. coli strains

*C. jejuni* strain 81-176-GFP was obtained by introduction via conjugation of plasmid pMA1 that contains the *gfp* gene encoding the green fluorescent protein (GFP) under control of the *C. jejuni metK* promoter [50]. *E. coli^inv^*-GFP strain was obtained by introduction of the same pMA1 plasmid via heat-shock transformation. *C. jejuni* strain 108-mCherry was constructed by introduction of plasmid pMA5-metK-mCherry via conjugation. This plasmid was constructed by replacing part of the *gfp* gene of pMA1-*metk*-*gfp* by the *mCherry* gene [51] of pTVmCherry (generously provided by J.M. Wells, Wageningen University) using the SphI and BstB1 restriction sites.

#### Construction of C. jejuni luciferase reporter strain

For construction of the MetK-Luciferase plasmid, the luciferase (*luc*) gene of *Photinus pyralis* was PCR amplified from the ConA Luc plasmid [52] using Phusion polymerase (Fermentas) with primers Luc-fwd and Luc-rev containing a SphI or SacI site (Table 1). The product was cloned into pJET1.2 (Invitrogen). The endogenous SphI and EcoRI sites in the cloned *luc* gene were then removed via nucleotide replacement without causing a change in amino acid sequence. Hereto PCRs were performed using the primer sets Luc-fwd and EcoRI-rev, and EcoRI-fwd and Luc-rev, respectively. The used EcoRI primers contained complementary sequences with the nucleotide exchange to remove the EcoRI site. The two PCR product fragments were mixed and amplified by PCR with primers Luc-fwd and Luc-rev to create the modified *luc* gene lacking the internal EcoRI site. The same strategy was applied to remove the SphI site except that this time the primers SphI-fwd and SphI-rev were used. The final product was ligated into pMA5-metK-GFP by replacing part of the *gfp* gene with the *luc* gene using the SacI and NcoI restriction sites, yielding pMA5-metK-luc. This plasmid was transformed into *E. coli* S17.1. The plasmid pMA5 is similar to the conjugative plasmid pMA1 [50] except that it lacks the 22 bp region containing the ribosomal binding site behind the *C. jejuni* metK promotor which remains active during *C. jejuni* infection of epithelial cells as suggested by proteome analysis [46]. The 22 bp region was removed by digestion of pMA1 with SphI and SacI and insertion at the same site of the metK promoter amplified from pMA1 with the primers metK-SphI-fwd and metK-SacI-rev. The nucleotide sequence of pMA5 and the modified *luc* gene were verified by DNA sequencing (Baseclear, Leiden).

**Table 1:** Primers used in this study.

Primer name	sequence
Luc-fwd	CCGAGCTCAGGAGATATCATGGAAGACGCCAAAAAC
Luc-rev	GGCCATGGTCACAATTTGGACTTTCCGCCC
EcoRI-fwd	GCACTGATAATGAACTCCTCTGGATCTACTGGG
EcoRI-rev	CCCAGTAGATCCAGAGGAGTTCATTATCAGTGC
SphI-fwd	GCGTCAGATTCTCGCACGCCAGAGATC
SphI- rev	GATCTCTGGCGTGCGAGAATCTGACGC
metK-SphI-fwd	GCATGC AGTTGATTTTAACTAACTTTTGCT
metK-SacI-rev	GAGCTC ATTTAAAATGAACCACAATTGTATC

Restriction site are underlined. Nucleotides used to delete restriction sites are in bold.

pMA5-metK-luc was introduced into *C. jejuni* strains via conjugation [50]. In short, a 16 h culture of *E. coli* S17-1 containing pMA5-metk-luc grown in LB medium was diluted to an optical density (550 nm) of 0.05 in 5 ml of LB medium without antibiotic and incubated shaking (160 rpm) at 37°C. In parallel an 16 h culture of *C. jejuni* was diluted to an optical density of 0.5 in 5 ml of HI medium and incubated under microaerophilic conditions (37°C, 160 rpm). When the recombinant *E. coli* culture reached an OD_550_ of 0.4, 1 ml of the *C. jejuni* suspension was collected by centrifugation (10 min, 5,000 *x g*) and resuspended in 1 ml of the *E. coli* culture. The mixture was suspended onto Mueller-Hinton plates and incubated (37°C) under microaerophilic conditions. After 5 h of incubation the bacteria were collected by centrifugation and plated onto saponin agar plates containing 4% lysed horse blood, CCDA *Campylobacter* selective supplement (Oxoid), and 50 µg/ml of kanamycin. Single *C. jejuni* colonies containing pMA5-metK-luc were collected after 48 h of incubation.

#### Gentamicin survival assay

Infection assays were performed with *C. jejuni* containing pMA5-metK-luc as described above, except that after 5 h of infection in Hepes buffer, cells were washed and replaced with DMEM containing gentamicin (250 µg/ml) to kill the extracellular *C. jejuni.* After 3 h of additional incubation in the presence of gentamicin, the cells were incubated (37°C, 5% CO_2_) in DMEM+10% FCS containing 50 µg/ml of gentamicin for up to 42 h. The amount of intracellular bacteria was estimated by treating the Caco-2 cells with 0.1% Triton X−100 in DMEM, (15 min, 20^o^C), followed by plating of serial dilutions of the cell lysate onto saponin agar plates containing kanamycin (50 µg/ml). Bacterial colony forming units were enumerated after 48 h of incubation of the saponin plates in a low oxygen (0.2%) or microaerophilic (5% oxygen) environment. Presented results are from three individual assays performed in duplicate. Data were analyzed using Graphpad Prism software.

#### Luciferase reporter assay

Infection experiments for luciferase and gentamicin killing assays were carried out simultaneously and in an identical fashion. Caco-2 cells were inoculated with *C. jejuni* and *C. jejuni* containing pMA5-metK-luc. After lysis of the cells with 0.1% Triton X−100 in DMEM, (15 min, 20^o^C), lysate was pelleted (10 min, 3,000 x *g*), further lysed with 1x RLB supplemented with 1% Triton−X100, and placed at −80°C for at least 30 min. The cell lysate was analyzed for luciferase activity in a luminometer (TD20/20, Turner Designs) immediately after adding 50 µl of Promega Luciferase Assay Agent to the sample as described [53]. Presented results are from three individual assays performed in duplicate. Data were analyzed using Graphpad Prism software.

## Supporting Information

Figure S1 **Effect of disruption of host cell cytoskeleton and ATP depletion on** ***C. jejuni*** **and** ***E. coli^inv^*** **invasion in semi-confluent int-407 cells.** Semi-confluent int-407 grown on 12 mm circular glass slides for 48 h in DMEM+5% FCS were pre-treated (1 h) with cytochalasin D (3 µM) and colchicine (10 µM) or Dinitrophenol (3 mM) and infected (2 h) with *C. jejuni* strain 108p4 (Red) and/or *E. coli^inv^* (Green). Cells were fixed and stained with WGA-Alexa fluor633 (Blue). Infected cells were visualized with confocal microscopy. As control, cells were pre-treated with an equivalent amount of solvent DMSO (Final concentration 0.2%) or solvent acetone (final concentration: 0.3%) and infected. Note the strong invasion of *C. jejuni* and the inhibition of *E. coli^inv^* invasion in the presence of the added compounds.
(TIF)

Figure S2 **Intracellular localization of** ***C. jejuni*** **within polarized Caco-2 islands.** Islands of Caco-2 were infected with *C. jejuni* strain 108 (red) for 1 h, washed, further incubated for up to 24 h, and fixed. After fixation, the cell surface was stained with WGA-Alexa fluor633 (Blue) (except when Lamp-1 was stained) and, after permeabilization, cellular compartments (Green) were stained with the marker antibodies EEA1 (early endosome), CD63 (endolysosome), or GM130 (Golgi apparatus) in combination with goat anti-mouse-Alexa fluor488. Infected cells were visualized with confocal microscopy. Results of the separate and merged channels are shown.
(TIF)

Figure S3 **Staining of intracellular** ***C. jejuni*** **with an** ***Campylobacter*** **-specific antibody at 48** **h of infection.** Islands of polarized Caco-2 cells were infected with *C. jejuni* strain 108 for 3 h in Hepes buffer, washed, incubated (3 h) with gentamicin (250 µg/ml) in DMEM, washed again, and incubated for an additional 42 h in DMEM+10% FCS with a low dose of gentamicin (50 µg/ml). After fixation cells were stained with WGA-alexa fluor633 (Blue). Confocal micrograph showing the presence of *C. jejuni* strain 108 (Red) as judged from the expression of mCherry (left panel) and after staining with *Campylobacter*-specific antibodies in combination with goat-anti-rabbit-Alexa fluor568 (Red)(right panel) at 48 h of infection.
(TIF)

Figure S4 **Determination of luciferase half-life in** ***C. jejuni*** **.** Chloramphenicol (40 µg/ml) was added to 2.5 ml of overnight culture of *C. jejuni* strain 108 containing pMA5-metK-luc and incubated (4 h) at 37°C in a 10% CO2 atmosphere. Every hour a sample of 2 x 10^8^ bacteria was taken and luciferase activity was determined and indicated as relative light units (RLU). RLU as expressed as percentage of total RLU measured at t = 0. Values are the mean of three separate experiments performed in duplo ± SEM. The half-life of luciferase in *C. jejuni* was determined as approximately 30 min.
(TIF)
