# Supplementary figures and images for: Basolateral Invasion and Trafficking of Campylobacter jejuni in Polarized Epithelial Cells
Source: PLoS One. 2013 Jan 28;8(1):e54759. doi: 10.1371/journal.pone.0054759 (PMC3557275; doi:10.1371/journal.pone.0054759)

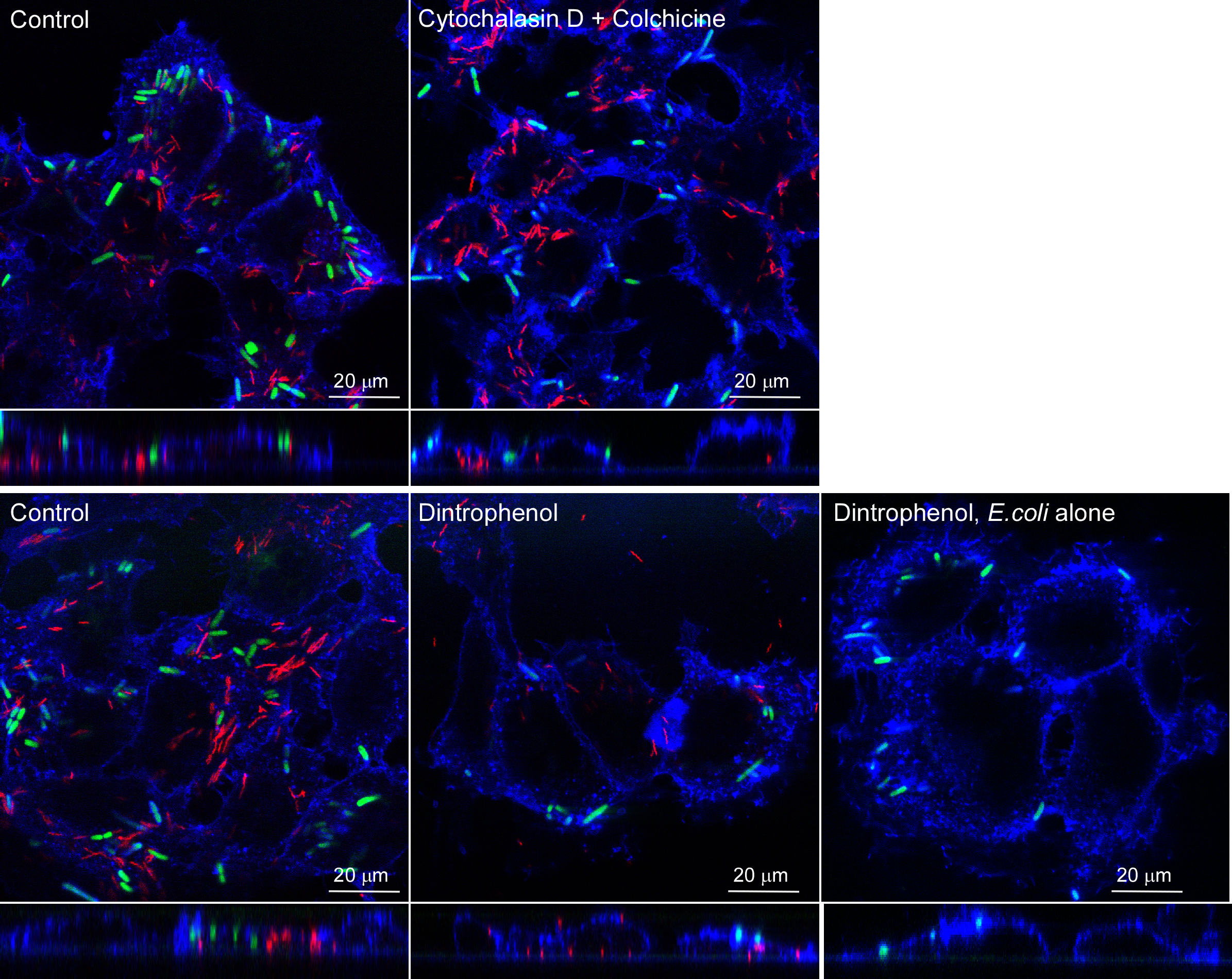

Supplement: Figure S1 — Effect of disruption of host cell cytoskeleton and ATP depletion on C. jejuni and E. coliinv invasion in semi-confluent int-407 cells. Semi-confluent int-407 grown on 12 mm circular glass slides for 48 h in DMEM+5% FCS were pre-treated (1 h) with cytochalasin D (3 µM) and colchicine (10 µM) or Dinitrophenol (3 mM) and infected (2 h) with C. jejuni strain 108p4 (Red) and/or E. coliinv (Green). Cells were fixed and stained with WGA-Alexa fluor633 (Blue). Infected cells were visualized with confocal microscopy. As control, cells were pre-treated with an equivalent amount of solvent DMSO (Final concentration 0.2%) or solvent acetone (final concentration: 0.3%) and infected. Note the strong invasion of C. jejuni and the inhibition of E. coliinv invasion in the presence of the added compounds. (TIF) [file pone.0054759.s001.tif]

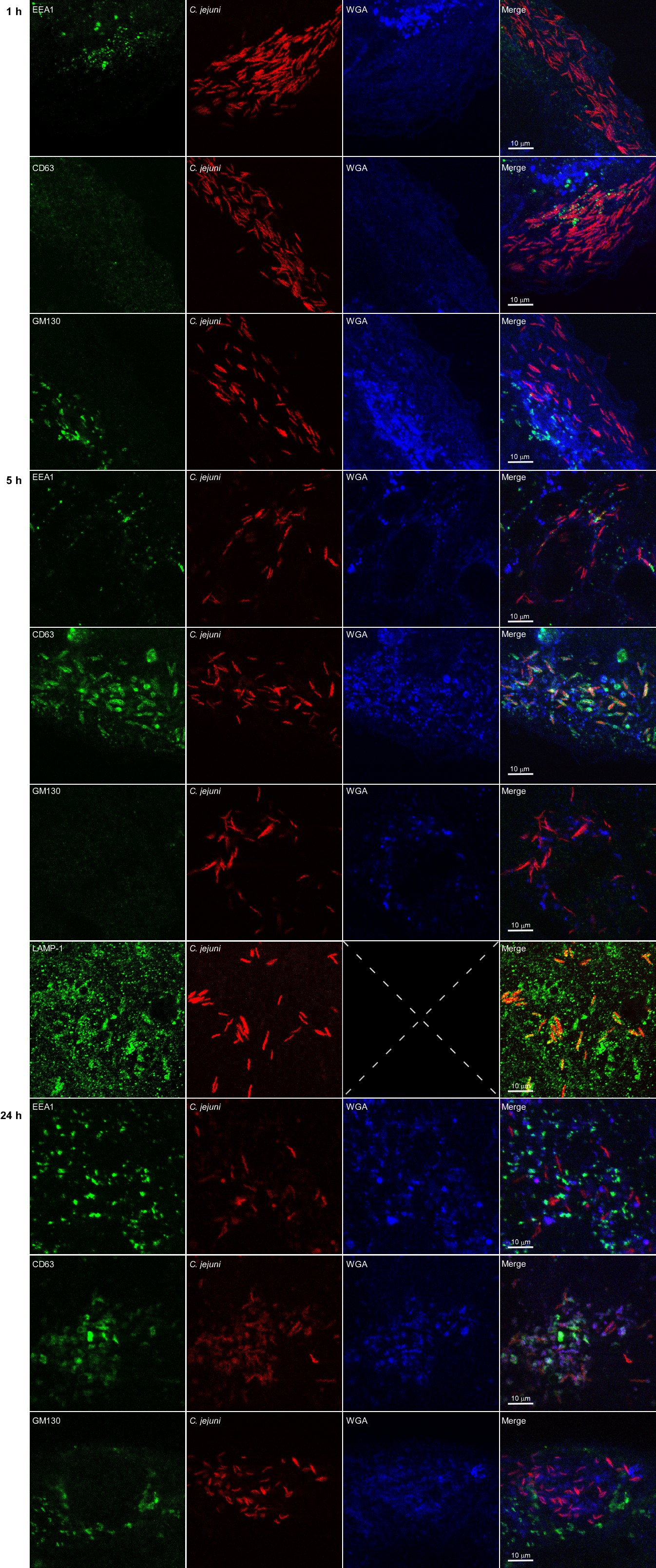

Supplement: Figure S2 — Intracellular localization of C. jejuni within polarized Caco-2 islands. Islands of Caco-2 were infected with C. jejuni strain 108 (red) for 1 h, washed, further incubated for up to 24 h, and fixed. After fixation, the cell surface was stained with WGA-Alexa fluor633 (Blue) (except when Lamp-1 was stained) and, after permeabilization, cellular compartments (Green) were stained with the marker antibodies EEA1 (early endosome), CD63 (endolysosome), or GM130 (Golgi apparatus) in combination with goat anti-mouse-Alexa fluor488. Infected cells were visualized with confocal microscopy. Results of the separate and merged channels are shown. (TIF) [file pone.0054759.s002.tif]

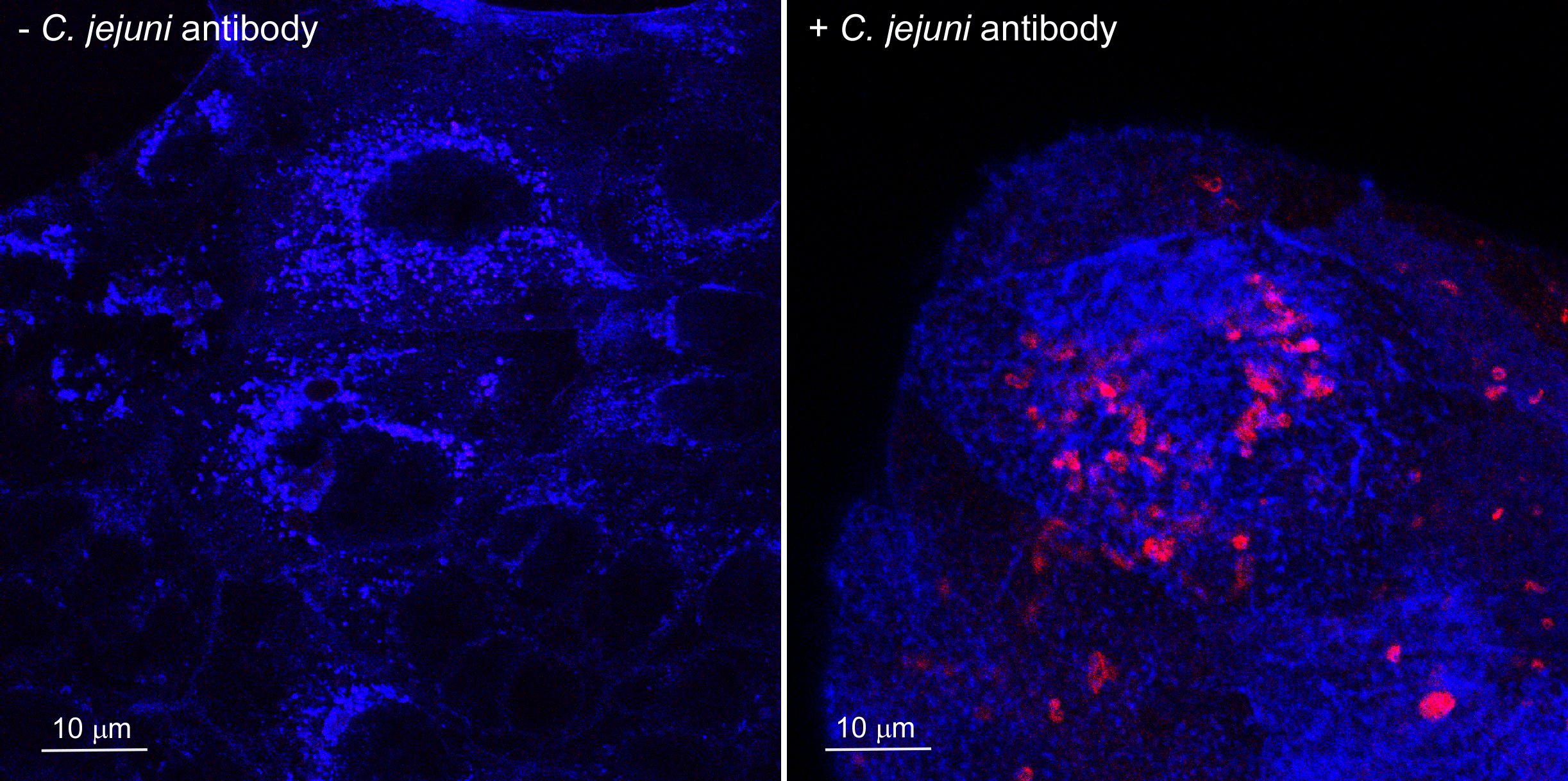

Supplement: Figure S3 — Staining of intracellular C. jejuni with an Campylobacter -specific antibody at 48 h of infection. Islands of polarized Caco-2 cells were infected with C. jejuni strain 108 for 3 h in Hepes buffer, washed, incubated (3 h) with gentamicin (250 µg/ml) in DMEM, washed again, and incubated for an additional 42 h in DMEM+10% FCS with a low dose of gentamicin (50 µg/ml). After fixation cells were stained with WGA-alexa fluor633 (Blue). Confocal micrograph showing the presence of C. jejuni strain 108 (Red) as judged from the expression of mCherry (left panel) and after staining with Campylobacter-specific antibodies in combination with goat-anti-rabbit-Alexa fluor568 (Red)(right panel) at 48 h of infection. (TIF) [file pone.0054759.s003.tif]
